# Effect of Flooding and the *nosZ* Gene in Bradyrhizobia on Bradyrhizobial Community Structure in the Soil

**DOI:** 10.1264/jsme2.ME16132

**Published:** 2017-06-08

**Authors:** Yuichi Saeki, Misato Nakamura, Maria Luisa T. Mason, Tsubasa Yano, Sokichi Shiro, Reiko Sameshima-Saito, Manabu Itakura, Kiwamu Minamisawa, Akihiro Yamamoto

**Affiliations:** 1 Faculty of Agriculture, University of MiyazakiMiyazaki 889–2192Japan; 2 Faculty of Life and Environmental Science, Shimane UniversityShimane 690–8504Japan; 3 College of Agriculture, Academic Institute, Shizuoka UniversityShizuoka 422–8529Japan; 4 Graduate School of Life Sciences, Tohoku UniversitySendai, Miyagi 980–8577Japan; 5 Center for Ecological Evolutionary Developmental Biology, Kyoto Sangyo UniversityKyoto 603–8555Japan; 6 College of Agriculture, Central Luzon State UniversityScience City of Muñoz, 3120 Nueva EcijaPhilippines

**Keywords:** *Bradyrhizobium*, community structure, denitrification, *nosZ*, flooded condition

## Abstract

We investigated the effects of the water status (flooded or non-flooded) and presence of the *nosZ* gene in bradyrhizobia on the bradyrhizobial community structure in a factorial experiment that examined three temperature levels (20°C, 25°C, and 30°C) and two soil types (andosol and gray lowland soil) using microcosm incubations. All microcosms were inoculated with *Bradyrhizobium japonicum* USDA6^T^, *B. japonicum* USDA123, and *B. elkanii* USDA76^T^, which do not possess the *nosZ* gene, and then half received *B. diazoefficiens* USDA110^T^wt (wt for the wild-type) and the other half received *B. diazoefficiens* USDA110Δ*nosZ*. USDA110^T^wt possesses the *nosZ* gene, which encodes N_2_O reductase; 110Δ*nosZ*, a mutant variant, does not. Changes in the community structure after 30- and 60-d incubations were investigated by denaturing-gradient gel electrophoresis and an image analysis. USDA6^T^ and 76^T^ strains slightly increased in non-flooded soil regardless of which USDA110^T^ strain was present. In flooded microcosms with the USDA110^T^wt strain, USDA110^T^wt became dominant, whereas in microcosms with the USDA110Δ*nosZ*, a similar change in the community structure occurred to that in non-flooded microcosms. These results suggest that possession of the *nosZ* gene confers a competitive advantage to *B. diazoefficiens* USDA110^T^ in flooded soil. We herein demonstrated that the dominance of *B. diazoefficiens* USDA110^T^wt within the soil bradyrhizobial population may be enhanced by periods of flooding or waterlogging systems such as paddy–soybean rotations because it appears to have the ability to thrive in moderately anaerobic soil.

The genus *Bradyrhizobium* is a group of soil bacteria that is active in symbiotic nitrogen fixation with soybeans and other leguminous plants. The nitrogen fixing ability of soybean bradyrhizobia depends on the strains present and environmental conditions involved in symbiosis (16, 32). The major nodulating rhizobia in soybean (*Glycine max.* [L.] Merr.) are *Bradyrhizobium japonicum*, *B. elkanii*, and *Sinorhizobium* (*Ensifer*) *fredii* (4, 14, 15, 17, 31, 45). The novel species, *B. diazoefficiens*, has been proposed as an independent species from *B. japonicum* (6), and *B. diazoefficiens* USDA110^T^ is referred to in this study rather than *B. japonicum* USDA110. Although other species of rhizobia have been implicated in soybean nodulation, the major endosymbionts in Japan are considered to be *B. japonicum*, *B. diazoefficiens*, and *B. elkanii* in acidic–neutral soils and *S. fredii* in alkaline soils (20, 23, 25, 26, 30, 39). Soybean-nodulating bacteria are widely distributed throughout the world, and endemism in their genetic diversity reflects the geographical and climatic differences as well as diversity of local soybean varieties. Therefore, analyses on the genetic diversity and distributions of indigenous soybean-nodulating rhizobia are important for improving our understanding of rhizobial ecology under various environmental conditions. Furthermore, an understanding of the functional relationships between different genetic types and environmental conditions will contribute to the more informed management of soybean production.

Denitrification ability, anaerobic respiration that reduces nitrate to nitrogen gas, also varies among strains of bradyrhizobia (28, 38). Some strains display complete denitrification activity and are able to release dinitrogen gas. Others show incomplete denitrification, which releases nitrous acid (NO_2_^−^), nitric oxide (NO), or nitrous oxide (N_2_O). Some strains appear to show no denitrification activity. Denitrification by soil bacteria generally requires four enzymes: periplasmic nitrate reductase (Nap), nitrite reductase (Nir), NO reductase (Nor), and N_2_O reductase (Nos). The respective genes that encode these enzymes are *napA*, *nirK*, *norCB*, and *nosZ* (2, 37). Nitrous oxide is an important greenhouse gas with a 100-yr global warming potential of 298 CO_2_ equivalents (12). Therefore, the mitigation of N_2_O release from the soybean rhizosphere, which is dominated by strains that display incomplete denitrification, is an important research task for sustainable soybean production (10, 11).

*Bradyrhizobium diazoefficiens* strain USDA110^T^ is known to have high nitrogen fixation activity, and is regarded as a useful rhizobial strain capable of inducing high soybean yield (9, 37). In addition, strain USDA110^T^ displays complete denitrification activity and may contribute to reducing the emission of N_2_O (13, 29). Therefore, USDA110^T^, with its high nitrogen fixation and complete denitrification activities, is expected to become an important rhizobial strain for sustainable soybean production. A thorough understanding of the physiological ecology of bradyrhizobia is important for ensuring the infection of soybean crops with useful *Bradyrhizobium* strains such as *B. diazoefficiens* USDA110^T^.

Our previous studies (23, 25–27) demonstrated that ecological niches for the bradyrhizobial community structure are arranged along a latitude gradient, suggesting that temperature is one of the important environmental factors in bradyrhizobial ecology. Furthermore, an analysis of the community structure of indigenous rhizobia showed that strains belonging to the Bj110 cluster, which is the cluster of USDA110^T^ based on a diversity analysis of the 16S–23S rRNA gene internal transcribed spacer (ITS) region, are abundant in silty soils such as fields converted from paddy rice production (26). Shiina *et al.* (33) also demonstrated that strains belonging to the cluster of USDA 6^T^ lacking the *nosZ* gene were dominant in andosols, whereas those belonging to the cluster of USDA 110^T^ possessing the *nosZ* gene were dominant in alluvial soils. These findings suggest that the endemism of bradyrhizobia depends on certain soil properties, and, thus, may affect denitrification activity through the presence or absence of strains that exhibit N_2_O reductase activity. Strains classified into those belonging to the Bj110 cluster possessing the *nosZ* gene may also be able to survive well in anaerobic soils with low redox potential, as typically occurs in paddy fields.

Based on previous findings (26, 33) showing that strains possessing the *nosZ* gene are abundant in fields converted from paddy rice fields, we hypothesize that the reducing conditions caused by waterlogging or flooding will be advantageous for strains that exhibit complete denitrification activity rather than those that are incapable of or only capable of incomplete denitrification. In order to test this hypothesis, we investigated the effects of water flooding on the community structure of bradyrhizobia at different temperatures and oxidation–reduction conditions in two different soils using microcosm incubations. Specifically, we compared differences in changes in the community structure over time with and without flooding in different soils with the USDA110^T^ strain possessing the *nosZ* gene or a mutant variant lacking the *nosZ* gene.

## Materials and Methods

The experiment was conducted with controlled microcosm incubations in a full factorial design involving two soil types (andosol and gray lowland soil), two flooding conditions (flooded or non-flooded), three temperature conditions (high, medium, and low), and two kinds of bradyrhizobial communities (the substitution of one out of four strains between two variants of the strain, one with the *nosZ* gene and one without). The response variable was the relative abundance of the various strains of added bradyrhizobia after a period of incubation. The community structure was assayed at 30 and 60 d, giving two incubation periods. All unique treatment level combinations were replicated three times. Details of the methods are stated henceforth.

### Soils and bradyrhizobia

Two different soils, an andosol (pH 6.28, EC 0.038 dS m^−1^, T-C: 4.63%, T-N: 0.34%, C/N ratio: 13.6) and gray lowland soil (pH 6.05, EC 0.102 dS m^−1^, T-C: 1.75%, T-N: 0.19%, C/N ratio: 9.4), were collected from the experimental fields of the University of Miyazaki, Japan. The soil groups to which the soils belonged were identified by the Classification of cultivated soils in Japan, Third approximation (5). Air-dried soil was passed through a sieve with a 2-mm screen, then sterilized by autoclaving three times at 121°C for 20 min with a 1-d interval between the second and third autoclave treatments. In each incubation, four *Bradyrhizobium* strains were used to create a bradyrhizobial community representative of those found in Japan (26). They were as follows: 1) *B. japonicum* strain USDA6^T^, which releases N_2_O as the end product of denitrification, 2) *B. japonicum* strain USDA123, which lacks any denitrification activity, 3) *B. elkanii* strain USDA76^T^, which releases NO_2_^−^ as the end product of denitrification, and 4) *B. diazoefficiens* strain USDA110^T^wt (wild-type), which releases N_2_ by complete denitrification (28), or *B. diazoefficiens* USDA110Δ*nosZ*, which is a mutant variant of USDA110^T^ that lacks an active N_2_O reductase gene (8).

### Preparation and incubation of microcosms

Each microbial strain stored on YMA slant medium (41) at 4°C used in the experiment was cultured for 5 d in 10 mL of YMB medium (41) at 145 rpm in a Multi Shaker (EYELA MMS-210, Tokyo Rikakikai, Tokyo, Japan) at 28°C in the dark. The cell density of each cultured strain was measured under a microscope by means of a hemocytometer for bacteria (Erma Inc., Tokyo, Japan) to achieve approximately 10^5^ cells g^−1^ (dry soil basis) for each strain, and each strain was mixed with prepared soil as a set of WT strains (WT set: USDA6^T^, 76^T^, 110^T^, and 123) or a set of a mutant and WT strains (Mut set: USDA6^T^, 76^T^, 110Δ*nosZ*, and 123). In the preparation of microcosms, USDA6^T^ was set as dominant for andosol and USDA110^T^ was set as dominant for gray lowland soil according to the previous study by Shiina *et al.* (33) that demonstrated the dominance of each strain depending on the soil type. The soil water content was adjusted to 40% of the maximum water-holding capacity with sterile distilled water. Potassium nitrate was added to microcosm soils at a rate of 50 mg NO_3_-N kg^−1^ dry soil. Microcosms were created by placing aliquots of 20 g (dry soil basis) of prepared soil into 50-mL sterile conical tubes with a gas-permeable filter cap (CELLSTAR, Cellreactor tube 50 mL with a Blue filter screw cap, Greiner Bio-One, Tokyo, Japan). Microcosms containing andosol or gray lowland soil were termed ADS or GLS microcosms, respectively. In non-flooded microcosms, soil was maintained at 40% water content of the maximum water-holding capacity. In flooded microcosms, soil was kept saturated with sterile distilled water with a free water content at 5 cm above the level of the soil surface. The three levels of incubation temperatures were 20°C (low), 25°C (medium), and 30°C (high) based on mean temperatures during rice paddy production in northern and southern regions of Japan. The opening of each microcosm was covered with a cap fit with a gas-permeable filter and incubations were performed in the dark in a temperature controlled incubator (TG-180-3LS, NK system, Nippon Medical & Chemical Instruments, Osaka, Japan). Microcosms were weighed and supplied with sterile distilled water every 3–4 d in non-flooded microcosms and every week in flooded microcosms to maintain the soil water condition. The oxidation–reduction potential (ORP) and dissolved oxygen content (DOC) were measured for representative microcosms of each treatment at intervals using a handheld multi-parameter meter (Intelligent Water Checker IWC-5, CUSTOM Corp., Tokyo, Japan) equipped with an ORP sensor (ORP-14 model, CUSTOM Corp) and handheld DO meter (Digital Oxygen DO-5509 model, SATO SHOUJI, Kawasaki, Japan). The ORP and DOC of non-flooded microcosms were assessed in 30-and 60-d incubation samples by the measurement of soil suspension (soil:water=1:4).

### Analysis of bradyrhizobial community structures

Soil samples of microcosms were sampled immediately after the preparation of microcosms (0 d) and after 30- and 60-d incubations, and stored at 20°C for later analyses. Environmental DNA (eDNA) was extracted from soil samples using an ISOIL DNA extraction kit (Nippon Gene, Tokyo, Japan) with the following modifications to the manufacturer’s procedure: Each sample was pre-treated with a bead beater homogenizer (Disruptor Genie, Scientific Industries, Bohemia, NY, USA) by mixing a 0.5-g soil sample with 950 μL of lysis solution HE (Nippon Gene), 50 μL of lysis solution 20S (Nippon Gene), and 0.5 g of glass beads (0.1 mm in diameter) for approximately 1 min. The following procedures were then conducted in accordance with the manufacturer’s instructions. The eDNA extract was purified using a polyvinylpolypyrrolidone spin-column (24). Polymerase chain reaction (PCR) and denaturing-gradient gel electrophoresis (DGGE) analyses were performed according to the methods described by Saeki *et al.* (24). PCR amplification targeting a part of the 16S–23S rRNA gene ITS region was conducted with *Ex-Taq* DNA polymerase (Takara Bio, Otsu, Japan), and a primer set for DGGE (DG-ITS-F: 5′-GTCCGCGAAACATCACTT-3′, DG-ITS-R-GC clump: 5′-CGCCCGCCGCGCCCCGCGCCCGTCCCGCCGCCCCCGCCCGGTCCACACACTCGGCAGA-3′) with extracted eDNA as the template. Touchdown PCR was performed as follows: pre-run at 94°C for 5 min; 30 cycles of denaturation at 94°C for 30 s, annealing at 68°C in the first cycle, which was lowered by 1°C per cycle through the tenth cycle, and at 58°C for the remaining 20 cycles for 30 s, then extension at 72°C for 30 s; and a final postrun at 72°C for 10 min. PCR amplicons were checked by 1.5% agarose gel electrophoresis in TAE buffer. As a negative control, we confirmed that no PCR amplicon was obtained from non-inoculated samples of the sterilized experimental soils. PCR amplicons were separated on an 8% polyacrylamide (2.6% C) gel with a 41–44% denaturing gradient at 60°C and 100 V for 14 h using the D-Code system (BioRad Laboratories, Hercules, CA, USA). After electrophoresis, the relative intensities of the visualized bands were measured using ImageJ program ver.1.48 (http://imagej.nih.gov/ij/) and their relative abundances were calculated from the peak area of each strain.

The abundance of each strain was obtained from the analysis of three replicates. A coded nomenclature was devised to describe each unique treatment combination. The first letter of the code was for soil: A for ADS microcosms or G for GLS microcosms. The second letter represented the water status: F for flooded or N for non-flooded. The third letter represented the alternate bradyrhizobia strains: W for microcosms containing the WT set with USDA110^T^wt or D for microcosms containing the Mut set with USDA110Δ*nosZ*. The fourth letter represented the incubation temperature: H for 30°C, M for 25°C, and L for 20°C. The incubation period was designated by appending 30 for 30 d and 60 for 60 d.

### Estimation of Community Structures

In order to evaluate the relative position among the community structure, we used the multi-dimensional scaling (MDS) method based on the Bray-Curtis similarity index (3, 19). We then used a cluster analysis to group the positions of the community structures based on the results of MDS. The Bray–Curtis similarity index is regarded as a robust index for reflecting the degree of similarities among communities (7). The Bray–Curtis similarity indices (*BC*) of all pair-wise comparisons were calculated using the following equation:

$$BC_{\text{AB}}=\Sigma |n_{\text{A}}-n_{\text{B}}|/(N_{\text{A}}+N_{\text{B}}),$$

where *BC*_AB_ is the dissimilarity between communities A and B, *n*_A_ and *n*_B_ represent the abundances of a given strain in communities A and B, respectively, and *N*_A_ and *N*_B_ represent the total abundance of all strains in communities A and B, respectively. MDS and cluster analyses were conducted using R software v3.2.1 (http://www.R-project.org/). The dendrogram for the cluster analysis was constructed by the Ward method, also using R. In addition, to evaluate the relationship between the abundances of the different bradyrhizobia and estimate environmental factors affecting the community structure, we used R to perform a principal-component analysis (PCA) on the abundance of the results of the microcosms in two separate analyses: one for microcosms with the WT set containing strains USDA6^T^, 76^T^, 110^T^wt, and 123, and the other for microcosms with the Mut set containing strains USDA6^T^, 76^T^, 110Δ*nosZ*, and 123.

### Statistical analysis

In the estimation of similarities among community structures in microcosms, statistical analyses were conducted using R software. In the comparison of two levels of treatments such as flooding conditions, Welch’s t-test was performed for each strain. In the comparison of three levels of treatments such as temperatures and incubation periods, Bonferroni’s significance test was performed for each strain. The effects of incubation factors, the water status, incubation temperatures, and incubation periods on the community structure were estimated with a multivariate analysis of variance (MANOVA) using Pillai’s trace statistics.

## Results

### Soil ORP and dissolved oxygen over time

**Fig. 1:**
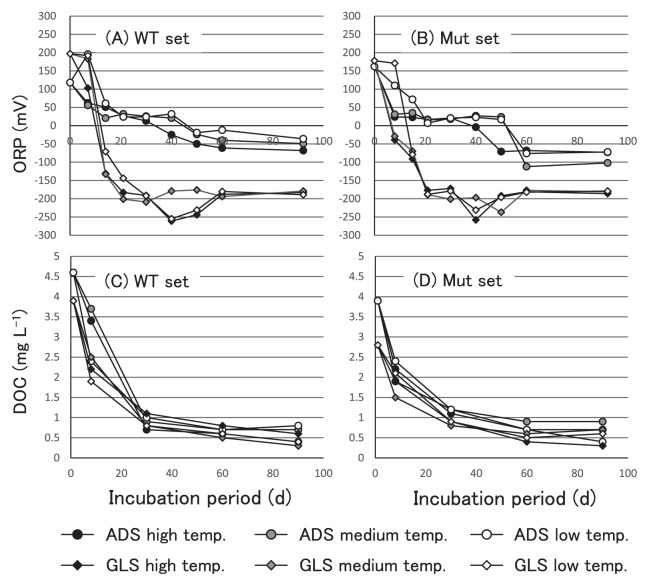
Oxidation–reduction potential, ORP (A and B) and dissolved oxygen content, DOC (C and D) in flooded microcosms incubated with each of two soil types (andosol and grey lowland soil) at each of three temperature conditions (low 20°C; medium 25°C, and high 30°C). Separate plots are shown for microcosms in which the terms WT set and Mut set contain strain sets, USDA6^T^, 76^T^, 110^T^wt, and 123 (A and C), or USDA6^T^, 76^T^, 110Δ*nosZ*, and 123 (B and D), respectively. Continuous significant differences in ORP values were detected between soil types from 14-d incubations (Welch’s t-test, *n*=3, *p*<0.01).

The soil ORP and DOC values of all non-flooded microcosms of andosol and gray lowland soil in 30- and 60-d soil samples indicated oxidative conditions such as 212±6.2 mV and 7.0±0.5 mg L^−1^, and 217±4.4 mV and 7.1±0.3 mg L^−1^ under the measurement conditions, respectively. On the other hand, all flooded microcosms initially had soil ORP values of approximately 200 mV, which then declined as the incubation time progressed before reaching equilibrium (Fig. 1). ORP values in GLS microcosms declined more rapidly and were approximately 100 mV at 14 d and stabilized at approximately −200 mV at 28 d. In ADS microcosms, ORP values were approximately +50 mV at 14 d and then stabilized at approximately −50 to −100 mV at 60 d. These changes over the course of the incubation period were similar to those for microcosms containing the WT set and Mut set. Thus, the only differences in ORP values during the incubation period were between ADS and GLS microcosms under flooded conditions. The DOC in flooded microcosm soils was similar among soil types and strains, at approximately 1 mg L^−1^ after a 30-d incubation and reaching stable values of approximately 0.3–0.9 mg L^−1^ from then until the end of the incubation period (Fig. 1). As supplementary data, pH and EC values were indicated in Table S1. Briefly, EC values decreased under flooded conditions in both soil types, suggesting a decrease in nitrate concentrations. On the other hand, pH values increased under flooded conditions.

### DGGE analysis of bradyrhizobial community structures

**Fig. 2:**
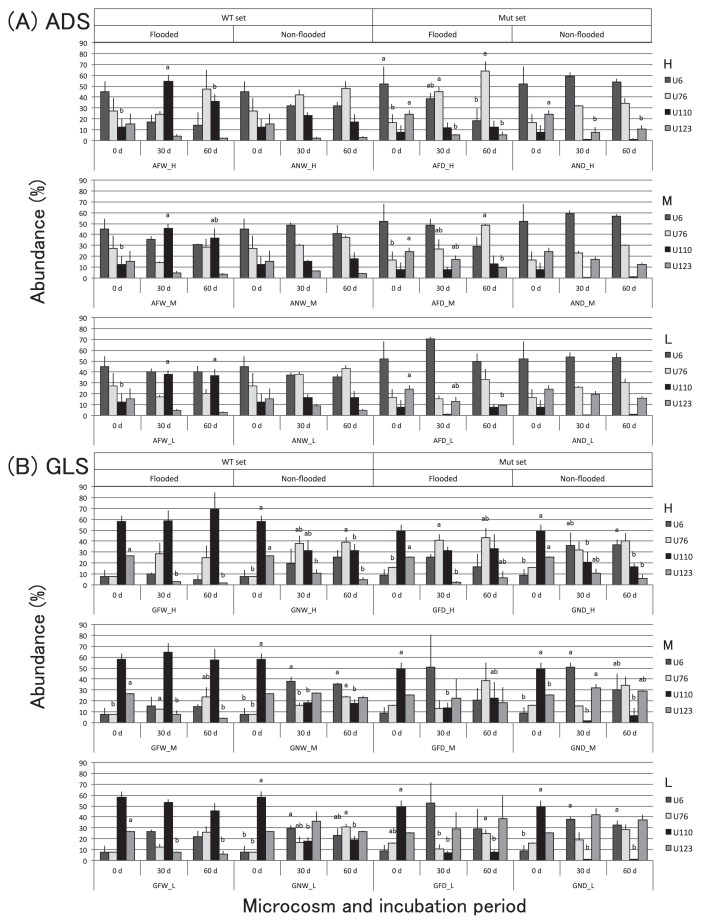
The relative abundance of each of four strains comprising the bradyrhizobial community structure in microcosms in an experiment examining combinations of two soil types (A: ADS and B: GLS), two soil moisture conditions (non-flooded, N, and flooded, F), and three temperature conditions (H: 30°C, M: 25°C, L: 20°C) after 0-, 30-, and 60-d incubations. Each value is the mean±standard deviation of three replicate microcosms (*n*=3). A significance test was conducted between incubation periods per strain per microcosm (Bonferroni, *p*<0.05). The four strains of *Bradyrhizobium* were *B. japonicum* USDA6^T^ (U6), *B. elkanii* USDA76^T^ (U76), *B. diazoefficiens* USDA110^T^ (U110), and *B. japonicum* USDA123 (U123). In half of the WT set microcosms (W), U110 was the USDA110^T^ wild-type strain possessing the *nosZ* gene, and in the other half of the Mut set (D), U110 was the USDA110Δ*nosZ* mutant that lacks the *nosZ* gene. The other three strains remained the same in all incubations. Microcosm nomenclature follows the abbreviations above, such that ANW_H indicates a microcosm with andosol, non-flooding condition, WT set, incubated at high temperature.

The population density of bradyrhizobia reached approximately 10^7^–10^8^ cells g^−1^ and became stable during the incubation period (Table S1). The DGGE analysis detected the four *Bradyrhizobium* strains as separate electrophoresed bands, and the strength of each band was regarded as an index of the abundance of each strain in the soil. The results of the DGGE analysis indicated significant changes in bradyrhizobial community structures during the incubation period in flooded and non-flooded microcosms (Fig. 2 and Table S2). In ADS microcosms with the WT set, very few changes in the relative abundance of the strains occurred in non-flooded microcosms, whereas the abundance of USDA110^T^wt increased significantly at all temperatures in flooded microcosms (Fig. 2A). In ADS microcosms with the Mut set, USDA110Δ*nosZ* remained at a low relative abundance in non-flooded and flooded microcosms. Strain USDA6^T^ remained in high abundance in non-flooded microcosms and slightly decreased in flooded microcosms though only significantly observed at higher incubation temperature. Meanwhile, USDA76^T^ slightly increased under higher temperatures regardless of the water status. In GLS microcosms with the WT set, changes in the community were as clearly detected as those in ADS microcosms (Fig. 2B). The relative abundance of USDA110^T^wt was initially high in flooded microcosms and remained so during the incubation period. Although strains USDA6^T^ and USDA76^T^ increased in flooded and non-flooded microcosms, their abundances were lower than those of USDA110^T^wt in flooded microcosms. The abundance of USDA110^T^wt significantly decreased from an initially high level in non-flooded microcosms. On the other hand, the abundance of USDA110Δ*nosZ* significantly decreased in flooded and non-flooded microcosms (except at a high temperature in flooded microcosms). USDA6^T^ and USDA76^T^ in GLS soil both slightly increased in flooded and non-flooded microcosms due to the low survivability of the mutant, similar to non-flooded microcosms with the WT set. USDA123 also reached a relatively high abundance in non-flooded low temperature incubations.

In comparisons of community structures in the 30-d and 60-d incubations under the same treatment, a significant difference was not observed in most community structures (Fig. 2 and Table S2). Since changes in the community structures of these experimental conditions appeared to reach stable phases in the 30-d incubation and maintained similar communities under the experimental conditions employed in the present study, further multivariate analyses, a cluster analysis and MDS analysis, were conducted for all data as set data for comparisons among all treatments and between soil types.

### Cluster analysis

**Fig. 3:**
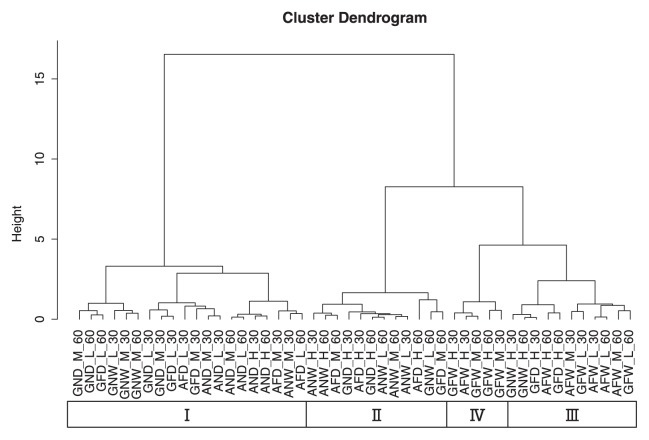
Dendrogram of bradyrhizobial community structures of microcosms in 30- and 60-d incubations. The dendrogram was constructed by the Ward method using Bray–Curtis Indices as distances among communities. Community nomenclature is a five-part name denoting soil type (A for ADS, G for GLS), the water status (N for non-flooded, F for flooded), bradyrhizobial strain variant (W for a wild-type possessing the *nosZ* gene and D for a mutant of the same strain that lacks the *nosZ* gene), temperature (H for 30°C, M for 25°C, and L for 20°C), and incubation period (30 for 30 d and 60 for 60 d); hence, ANW_H_30 indicates a microcosm with andosol, non-flooding conditions, the WT set, incubated at a high temperature for 30 d.

**Fig. 4:**
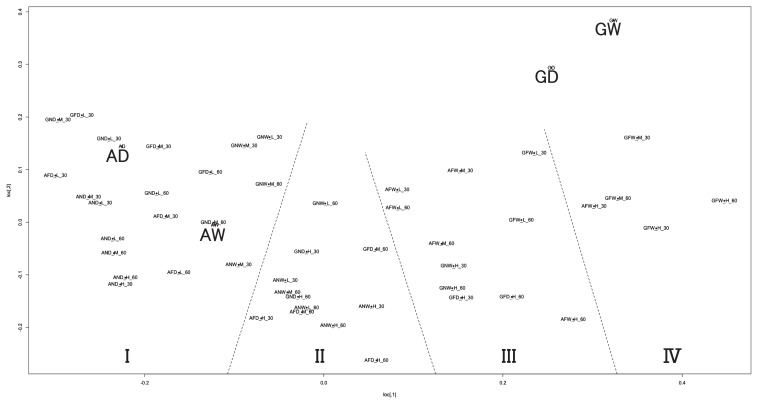
Two-dimensional–multidimensional-scaling (2D–MDS) plots of bradyrhizobial community structures of all microcosms in 0-, 30-, and 60-d incubations. Community nomenclature is a five-part name denoting soil type (A for ADS, G for GLS), the water status (N for non-flooded, F for flooded), bradyrhizobial strain variant (W for a wild-type possessing the *nosZ* gene and D for a mutant of the same strain that lacks the *nosZ* gene), temperature (H for 30°C, M for 25°C, and L for 20°C), and incubation period (30 for 30 d and 60 for 60 d). Clusters I, II, III, and IV correspond to those discriminated by the dendrogram in Fig. 3. The positions denoted by AW, AD, GW, and GD are MDS plots for the microcosms of different soil types (A or G) by bradyrhizobial strain variant (W or D) in a 0-d incubation.

The dendrogram based on the similarity indices of community structures in 30- and 60-d incubations indicated four major clusters: I, II, III, and IV (Fig. 3). Cluster numbers were decided from the ordering of the clusters in the MDS plots (Fig. 4). Based on strain abundance from Fig. 2, cluster I was comprised of communities in which USDA6^T^ and USDA76^T^ were dominant in microcosms with some WT sets in non-flooded and most Mut sets. Cluster I, having the lowest similarity index values, was strongly divided from the other clusters. Cluster IV was comprised of communities in which USDA110^T^wt was dominant in flooded microcosms. Cluster II was comprised of communities in which the dominant strains were predominantly USDA6^T^ and USDA76^T^ in non-flooded microcosms with 110^T^wt. Cluster III was mostly comprised of flooded microcosms and was closely related to cluster IV.

### MDS analysis of bradyrhizobial community structures

**Fig. 5:**
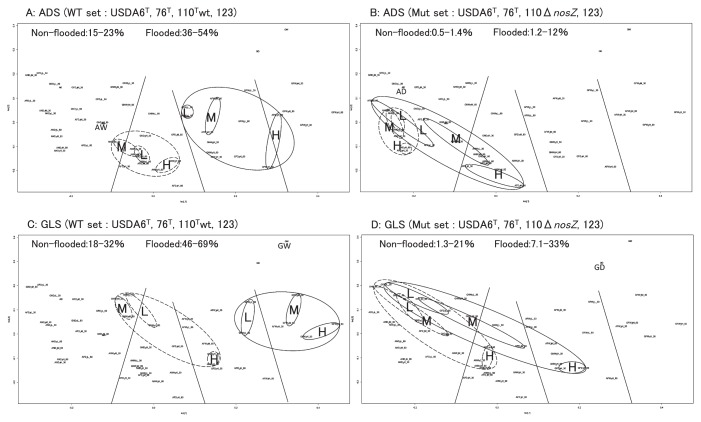
Differences in community structures between microcosms under flooding conditions (ellipses with solid lines) and non-flooding conditions (ellipses with dashed lines) within the 2D–MDS space. The percentages of “flood” or “non-flood” refer to the range of abundance (%) of the *Bradyrhizobium diazoefficiens* USDA110 strain in the 2D–MDS ellipse space for the respective microcosms being characterized. The ellipses denoted by H, M, or L indicate the characteristic 2D–MDS space for the respective subsets of microcosms of high, medium, and low incubation temperatures. The four panels are the analysis of the characteristic space for microcosms containing andosol (A and B) or gray lowland soil (C and D) and the WT set (A and C) or Mut set (B and D) containing variant strains of *B. diazoefficiens* within the bradyrhizobial community structure.

MDS plots of the community structures of the different microcosms were classified into four clusters: I, II, III, and IV, based on the criteria that formed the clusters in the dendrogram (Fig. 4). Flooded microcosms containing the WT sets formed an apparently independent cluster from non-flooded microcosms and microcosms with the Mut sets, regardless of the soil type (Fig. 5A and C). Microcosms with the Mut sets overlapped with flooded and non-flooded microcosms, regardless of the soil type (Fig. 5B and D). Community structures in flooded microcosms containing Mut set strains displayed a wider distribution in the MDS space than those with the non-flooded Mut set, with differentiation being more pronounced in flooded and high temperature incubations.

### PCA of bradyrhizobial community structures

**Fig. 6:**
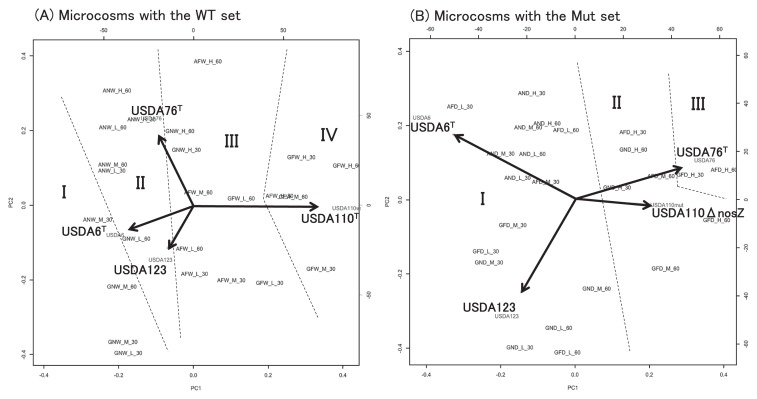
Principal-component analysis of all community structures in 30- and 60-d incubations separated into microcosms according to whether the *Bradyrhizobium diazoefficiens* strain was USDA110^T^wt (A) or the USDA110Δ*nosZ* mutant (B). Numbers on PCA plots are cluster numbers based on the dendrogram from the cluster analysis. Arrows are the percentage contributions of each strain affecting the community structures.

The results of PCA on bradyrhizobial community structures are shown in Fig. 6. A high cumulative proportion of variance was contained within the first two principal components (65% on PC1 and 23% on PC2 for WT sets and 64% on PC1 and 26% on PC2 for Mut sets), and most of the relationships among the four strains under the experimental conditions used in this study were explained using 2D-PCA plots. Strains USDA6^T^ and USDA76^T^ became dominant under the aerobic conditions of non-flooded microcosms, whereas USDA110^T^wt became dominant under the reducing conditions of flooded treatments. As a result, a high proportion of dominance under the reducing conditions was observed with USDA110^T^wt, which was detected in cluster IV. In contrast, since USDA110Δ*nosZ* showed decreased abundance in flooded microcosms, the proportion of variance observed with USDA110Δ*nosZ* was markedly lower than that of the WT strain. Most communities were detected in cluster I, whereas no communities belonging to cluster IV were detected. These results were supported by the results of MANOVA with high F values for the effect of flooded conditions in the WT set and relatively low values in the Mut set (Table 1). Additionally, USDA76^T^ increased its abundance at higher temperatures, whereas USDA123 slightly increased its abundance at lower temperatures (Fig. 2 and Table S2). Thus, a negative correlation was detected between strains USDA76^T^ and USDA123 on PC2 (Fig. 6A). Based on the results of MANOVA, the effects of temperature on community structures were weaker than those of flooded conditions in this study (Table 1).

## Discussion

In the present study, we attempted to demonstrate the effects of strains possessing the *nosZ* gene on bradyrhizobia community structures in microcosm soils under different temperatures and oxidation-reduction conditions.

Community structures in flooded and non-flooded microcosms with the WT set, containing strains USDA6^T^, 76^T^, 110^T^wt, and 123, displayed shifts for similar community structures in ADS and GLS soil types. Changes in community structures in WT set microcosms that were flooded were more pronounced than in those that were not flooded. USDA110^T^wt, which is *nosZ* positive (*nosZ*+), became dominant under the anaerobic conditions in flooded microcosms (Fig. 2). In flooded microcosms, the dominance of USDA110^T^wt was more pronounced in GLS than in ADS microcosms. In Mut set microcosms, USDA110Δ*nosZ*, lacking *nosZ* (Δ*nosZ*), did not become dominant in any of the flooded microcosms, and community structures with this Δ*nosZ* mutant displayed similar changes under flooded and non-flooded conditions. Although dissolved oxygen concentrations were not significantly different among flooded microcosms, ORP values decreased more rapidly in GLS than in ADS, and significantly decreased before 14 d of incubation (Fig. 1). Although the reason for the difference in ORP changes between soil types currently remains unclear, a previous study reported that the difference in ORP changes was affected by soil types, and soil that developed from silt showed rapid decreases in ORP under flooded conditions (42). Relationships among nitrate, pH, and EC have been reported by many researchers. Positive correlations have been reported between nitrate concentrations and EC values, and negative correlations between pH and nitrate indicate (21). Based on EC and pH values, denitrification may have occurred during the incubation period. Denitrification in soil is expressed as ORP values in the range of approximately ±100 mV, and the optimal range of values to promote denitrification is ±50 mV (44). Therefore, denitrification by bradyrhizobia in flooded GLS microcosms may have occurred and reached a peak after approximately 14 d of incubation, and denitrification activities may have decreased until approximately 30 d of incubation. In flooded ADS microcosms, denitrification by bradyrhizobia may have occurred later than in GLS microcosms and may then have been expressed for most of the incubation period because ORP values remained in the optimal range for denitrification. These results suggest that the ORP and DOC values reached were in a range that led to changes and differentiation in bradyrhizobial community structures in different soil types. We previously indicated that temperature is one of the factors affecting the construction of bradyrhizobial community structures in soils in a microcosm study (24). The present results also suggest that soil ORP is an environmental factor affecting the ecology and occupancy of bradyrhizobia in soils.

The cluster analysis and MDS analysis indicated that all microcosms in cluster IV were flooded and contained USDA110^T^wt as the dominant strain (Fig. 2, 3, and 4). Furthermore, cluster I mostly comprised microcosms with USDA110Δ*nosZ* (AND, AFD, GND, and GFD) and GNW with USDA110^T^wt (Fig. 3), and was relatively well separated from clusters mostly comprised of flooded microcosms containing USDA110^T^wt (clusters III and IV). As shown in Fig. 5, the positions of the different community structure clusters were similar between ADS and GLS microcosms, with the greatest influence shown by flooding and USDA110^T^ deployed. The slight difference observed in the positions of the different community structures between ADS and GLS microcosms may be due to different ORP profiles in the two soils. In microcosms with the Mut set, differences in changes in community structures were not readily distinguishable between flooded and non-flooded microcosms, particularly for the lower temperature incubations (Fig. 5B and D). However, higher temperatures had a stronger influence on the separation of community structures in microcosms with the Mut set under flooded conditions than under lower temperatures and non-flooded conditions. This result suggests that other functions that were influenced by temperature, except for *nosZ*, function in the differentiation of community structures under flooded conditions. Sameshima-Saito *et al.* (29) reported that symbiotic USDA110^T^, indicating the final step in denitrification activity from N_2_O to N_2_, reduces N_2_O surrounding the soybean root system. Additionally, the present results suggest that the ability to undertake the final step in denitrification activity is an advantage to strains that possess the *nosZ* gene, and these strains will outperform strains in flooded soil that do not possess this gene, thereby altering the bradyrhizobial community structure. According to the study by Thauer *et al.* (40), total energy acquisition by complete denitrification from nitrate to N_2_ gas is 1120.9 kJ, and partial energy acquisition from N_2_O to N_2_ is 341.4 kJ. Energy acquisition by complete denitrification activity containing N_2_O reductase is higher by approximately 30% than that of incomplete denitrification until N_2_O. This difference in energy acquisition by denitrification may be a competitive advantage to bradyrhizobia possessing the *nosZ* gene in flooded soils. Differences between soil types in the community structure response to anaerobic conditions may be attributable to differences in changes in ORP values after flooding. Temperature is also an important factor in the establishment of bradyrhizobial community structures, and will always need to be considered when verifying the effects of the expression of denitrification-related genes on community structures. The PCA of bradyrhizobial community structures showed a strong negative correlation between strains USDA6^T^ and USDA110^T^wt on PC1 (Fig. 6A). Although this negative correlation was also detected between USDA6^T^ and USDA110Δ*nosZ*, the relative contribution of USDA110Δ*nosZ* to total variance was markedly less than that of USDA110^T^wt (Fig. 6B). These results suggest that a negative correlation may exist due to the interaction between strains USDA110^T^ (*nosZ*+) and USDA6^T^ (*nosZ*−). Shiina *et al.* (33) reported that *nosZ*− strains were dominant in andosols and *nosZ*+ strains were dominant in alluvial soils in Japan. Our previous study (26) also detected a high abundance of strains belonging to the USDA110^T^ cluster in gray lowland and gley soils in Japan, indicating that flooding and poor drainage are important environmental factors favoring bradyrhizobia strains possessing the *nosZ* gene. These results were supported by those of MANOVA (Table 1). The effects of flooded conditions on the bradyrhizobial community structures of WT sets were stronger than those of other environmental factors in the present study. On the other hand, the effects of flooded conditions on the community structures of the Mut sets were weaker than those of the WT sets. Furthermore, GLS microcosms may be affected more by flooded conditions than ADS microcosms due to differences in the ORP status. Additionally, there was a negative correlation between strains USDA76^T^ and USDA123 on PC2 (Fig. 6A). This negative correlation may have been induced by temperature during the incubation. USDA76^T^ was slightly more dominant at higher temperatures, and USDA123 at lower temperatures. This is in accordance with the known geographical distribution of bradyrhizobia strains in Japan and the United States (26, 27, 35). Furthermore, many studies have suggested that the relationship between genomic diversity and the field distribution of indigenous soybean-nodulating rhizobia is affected by soil environmental properties such as temperature being influenced by latitude and altitude, phosphorus content, EC, and soil pH (1, 18, 22, 34, 36, 43). The present results also indicate that the mechanism of dominance in poorly aerated soils under field conditions is related to the denitrification activity of bradyrhizobia. Since ORP values less than −200 mV are indicative of completely anaerobic conditions that induce methane fermentation, paddy fields with ORP values in the range of ±100 mV, and particularly in the range of ±50 mV (44), present optimum field conditions for the dominance of bradyrhizobia possessing denitrification activity, such as *nosZ*+ bradyrhizobial strains. Bradyrhizobia that have incomplete denitrification will become slightly more dominant in aerobic soils, even those in fields converted from paddy to upland cropping for an extended period. The results of MANOVA suggest that the change in ORP caused by soil saturation has a greater effect on bradyrhizobial community structures than temperature changes.

In the present study, we demonstrated that the soil water status is one of the important environmental factors affecting soil bradyrhizobial community structures. The ORP status appears to be an environmental factor that has a greater impact on bradyrhizobial community structures than temperature, at least in the range of the temperatures examined in the present study. Investigations on bradyrhizobial community structures not only under a wider range of temperatures, but also with a wide range of various environmental factors are needed in order to elucidate rhizobial ecological traits. Furthermore, other microorganisms may be concerned with the establishment of bradyrhizobial community structures. Further studies are needed in order to elucidate rhizobial ecology including other soil microorganisms. *B. diazoefficiens* USDA110^T^ has not only a strong nitrogen fixation ability, but also the ability to undertake complete denitrification (28). Strains with these abilities may make an important contribution to sustainable soybean production (13). Our results suggest that bradyrhizobial community structures are manipulated to favor or maintain useful strains including *B. diazoefficiens* USDA110^T^ through soil management that involves periodic water flooding, such as the soybean–paddy rotation system. Further investigations on the ecological functions of bradyrhizobial communities that lead to control by agricultural management are important for the continued development of sustainable soybean production.

## Supplementary material



## Figures and Tables

**Table 1:** Results of a multivariate analysis of variance (Pillai’s trace) on community structures in microcosms.

	ADS WT set	ADS Mut set	GLS WT set	GLS Mut set
Flood condition (F)	*F* (1, 34)=28.03, *p*=4.42e-09	*F* (1, 34)=9.09, *p*=0.000169	*F* (1, 34)=36.07, *p*=2.22e-10	*F* (1, 34)=4.50, *p*=0.00961
Temperature (T)	*F* (2, 33)=4.12, *p*=0.00148	*F* (2, 33)=3.40, *p*=0.00561	*F* (2, 33)=3.71, *p*=0.00319	*F* (2, 33)=5.01, *p*=0.000292
Incubation period (IP)	*F* (1, 34)=4.96, *p*=0.00614	*F* (1, 34)=2.71, *p*=0.0612	*F* (1, 34)=1.65, *p*=0.198	*F* (2, 34)=5.29, *p*=0.00445
F×T	*F* (5, 30)=7.97, *p*=4.45e-11	*F* (5, 30)=5.11, *p*=3.49e-07	*F* (5, 30)=8.99, *p*=2.56e-12	*F* (5, 30)=3.03, *p*=0.000580
F×IP	*F* (3, 32)=5.91, *p*=1.54e-06	*F* (3, 32)=3.64, *p*=0.000610	*F* (3, 32)=4.83, *p*=2.51e-05	*F* (3, 32)=3.10, *p*=0.00259
T×IP	*F* (5, 30)=2.86, *p*=0.00106	*F* (5, 30)=2.32, *p*=0.00751	*F* (5, 30)=2.01, *p*=0.0224	*F* (5, 30)=3.24, *p*=0.000271
F×T×IP	*F* (11, 24)=6.30, *p*=3.94e-11	*F* (11, 24)=3.58, *p*=3.23e-06	*F* (11, 24)=5.20, *p*=2.70e-09	*F* (11,24)=2.18, *p*=0.00300
