# Supplementary material for: Effect of Flooding and the nosZ Gene in Bradyrhizobia on Bradyrhizobial Community Structure in the Soil
Source: Microbes Environ. 2017 Jun 8;32(2):154–63. doi: 10.1264/jsme2.ME16132 (PMC5478539; doi:10.1264/jsme2.ME16132)
Supplement: Supplementary file 1 [file 32_154_s1.pdf]

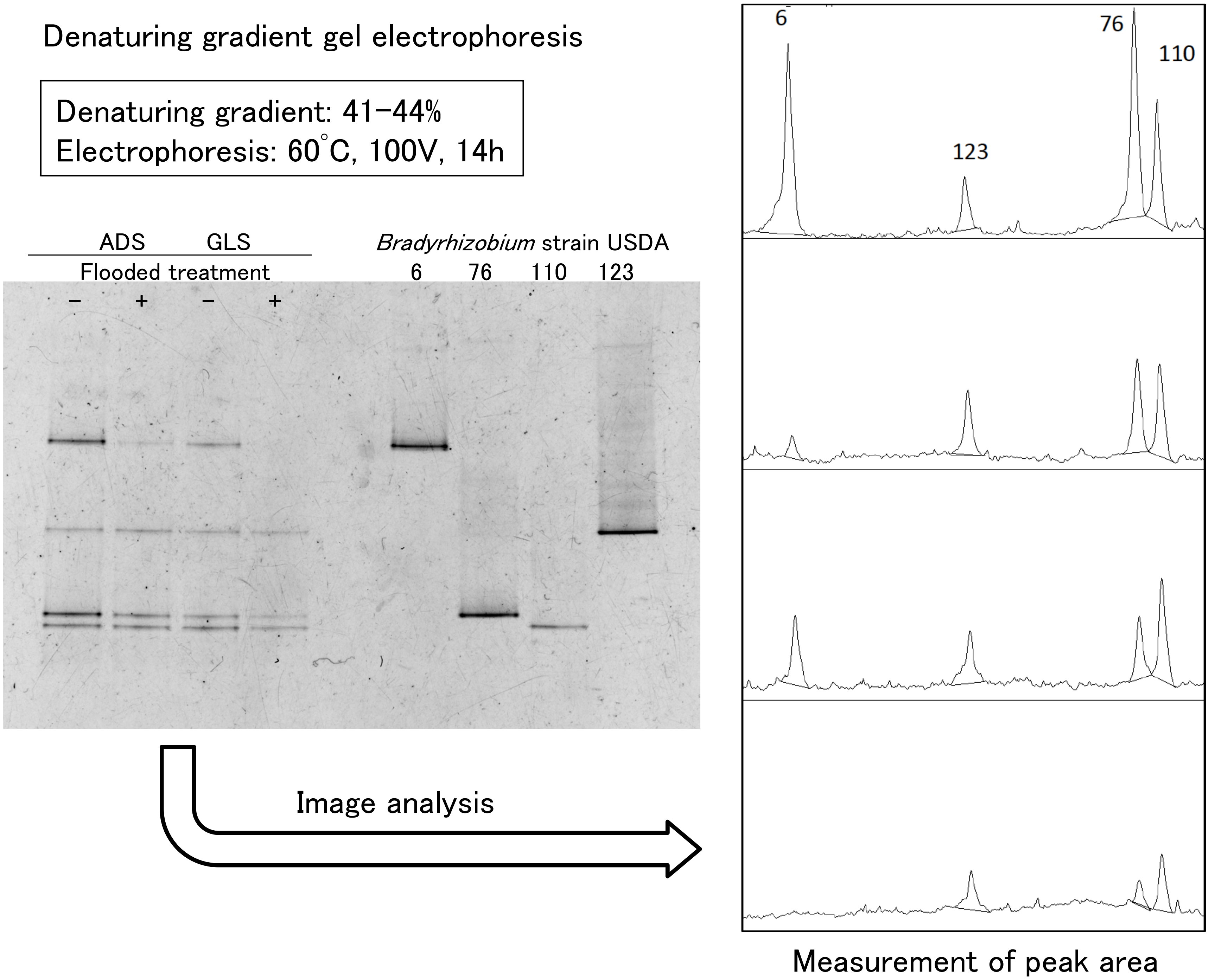

Fig. S1. Schematic representation of a DGGE analysis and the image analysis procedure for the estimation of community structures.

Table S1 pH, EC values, and population densities of bradyrhizobia in soil microcosms.

	pH(H_2O) 1:5 extract*			EC (dS m^{-1}) 1:5 extract*			Population density (CFU g^{-1} dry soil)		
	0 day	30 day	60 day	0 day	30 day	60 day	0 day	30 day	60 day
ANW_H	5.93 ± 0.02 a	6.09 ± 0.05 ab	6.05 ± 0.00 b	0.38 ± 0.00 a	0.28 ± 0.02 b	0.33 ± 0.01 b	(1.26 ± 0.45) × 10^{6}	(3.17 ± 0.28) × 10^{7}	(3.64 ± 1.30) × 10^{7}
ANW_M		6.06 ± 0.01 b	6.05 ± 0.02 b		0.31 ± 0.01 b	0.35 ± 0.01 a		(3.09 ± 1.25) × 10^{7}	(3.81 ± 2.09) × 10^{7}
ANW_L		6.11 ± 0.07 ab	6.05 ± 0.00 b		0.31 ± 0.04 a	0.34 ± 0.02 a		(3.17 ± 1.56) × 10^{7}	(8.38 ± 1.22) × 10^{6}
AFW_H	5.93 ± 0.02 a	6.80 ± 0.13 b	6.78 ± 0.05 b	0.38 ± 0.00 a	0.21 ± 0.05 ab	0.19 ± 0.00 b	(1.26 ± 0.45) × 10^{6}	(1.04 ± 0.56) × 10^{8}	(1.09 ± 0.39) × 10^{7}
AFW_M		6.65 ± 0.02 b	6.74 ± 0.08 b		0.18 ± 0.00 b	0.18 ± 0.00 b		(1.27 ± 0.30) × 10^{8}	(3.85 ± 0.80) × 10^{7}
AFW_L		6.81 ± 0.18 b	6.63 ± 0.02 b		0.17 ± 0.01 b	0.16 ± 0.01 b		(9.83 ± 1.84) × 10^{7}	(1.18 ± 1.62) × 10^{7}
AND_H	6.49 ± 0.02 a	6.21 ± 0.19 ab	6.33 ± 0.04 b	0.41 ± 0.02 a	0.37 ± 0.01 b	0.32 ± 0.02 b	(8.82 ± 3.29) × 10^{5}	(1.04 ± 0.33) × 10^{8}	(4,72 ± 1.09) × 10^{7}
AND_M		6.13 ± 0.04 b	6.19 ± 0.03 b		0.40 ± 0.01 a	0.31 ± 0.03 a		(1.04 ± 0.11) × 10^{8}	(5.48 ± 0.76) × 10^{7}
AND_L		6.18 ± 0.05 b	6.17 ± 0.01 b		0.42 ± 0.07 ab	0.31 ± 0.01 b		(1.27 ± 0.75) × 10^{8}	(8.20 ± 1.16) × 10^{7}
AFD_H	6.49 ± 0.02 a	6.92 ± 0.05 b	6.98 ± 0.04 b	0.41 ± 0.02 a	0.25 ± 0.01 b	0.23 ± 0.01 b	(8.82 ± 3.29) × 10^{5}	(2.76 ± 0.95) × 10^{8}	(7.77 ± 4.44) × 10^{7}
AFD_M		6.94 ± 0.03 b	6.92 ± 0.16 a		0.25 ± 0.01 b	0.22 ± 0.01 b		(1.37 ± 0.25) × 10^{8}	(8.76 ± 1.68) × 10^{7}
AFD_L		6.95 ± 0.04 b	7.20 ± 0.05 c		0.24 ± 0.01 b	0.26 ± 0.01 b		(1.14 ± 0.27) × 10^{8}	(1.01 ± 0.82) × 10^{8}
GNW_H	6.09 ± 0.02 a	5.88 ± 0.03 b	5.95 ± 0.02 c	0.28 ± 0.01 a	0.32 ± 0.04 a	0.33 ± 0.03 a	(1.33 ± 1.00) × 10^{6}	(3.81 ± 1.28) × 10^{7}	(4.78 ± 1.76) × 10^{7}
GNW_M		6.00 ± 0.07 a	5.89 ± 0.12 a		0.33 ± 0.01 a	0.28 ± 0.04 a		(6.76 ± 2.36) × 10^{7}	(3.26 ± 1.44) × 10^{7}
GNW_L		5.99 ± 0.02 b	5.96 ± 0.03 b		0.26 ± 0.03 a	0.31 ± 0.08 a		(5.59 ± 0.43) × 10^{7}	(3.25 ± 1.65) × 10^{7}
GFW_H	6.09 ± 0.02 a	6.51 ± 0.21 ab	6.42 ± 0.06 b	0.28 ± 0.01 a	0.13 ± 0.01 b	0.16 ± 0.03 b	(1.33 ± 1.00) × 10^{6}	(1.43 ± 0.25) × 10^{8}	(1.06 ± 1.21) × 10^{8}
GFW_M		6.68 ± 0.10 b	6.20 ± 0.08 a		0.14 ± 0.02 b	0.12 ± 0.00 b		(9.24 ± 1.74) × 10^{7}	(9.81 ± 5.87) × 10^{7}
GFW_L		6.53 ± 0.11 ab	6.28 ± 0.05 b		0.12 ± 0.01 b	0.12 ± 0.08 b		(1.64 ± 0.51) × 10^{8}	(1.83 ± 1.41) × 10^{7}
GND_H	6.09 ± 0.02 a	6.01 ± 0.07 a	5.99 ± 0.06 a	0.28 ± 0.01 a	0.35 ± 0.04 a	0.34 ± 0.05 a	(1.13 ± 0.95) × 10^{6}	(4.81 ± 1.78) × 10^{7}	(3.65 ± 0.78) × 10^{7}
GND_M		5.96 ± 0.09 a	6.01 ± 0.03 a		0.34 ± 0.02 a	0.33 ± 0.03 a		(6.26 ± 3.66) × 10^{7}	(3.61 ± 0.55) × 10^{7}
GND_L		5.85 ± 0.02 b	6.10 ± 0.10 ab		0.36 ± 0.05 a	0.28 ± 0.02 a		(5.60 ± 0.34) × 10^{7}	(3.16 ± 0.74) × 10^{7}
GFD_H	6.09 ± 0.02 a	6.69 ± 0.05 c	6.28 ± 0.04 b	0.28 ± 0.01 a	0.18 ± 0.01 b	0.16 ± 0.00 b	(1.13 ± 0.95) × 10^{6}	(1.03 ± 0.61) × 10^{8}	(7.22 ± 0.50) × 10^{7}
GFD_M		6.53 ± 0.13 a	6.42 ± 0.21 a		0.17 ± 0.01 b	0.16 ± 0.01 b		(8.43 ± 0.49) × 10^{7}	(6.73 ± 0.60) × 10^{7}
GFD_L		6.57 ± 0.09 b	6.33 ± 0.10 ab		0.16 ± 0.00 b	0.16 ± 0.01 b		(1.87 ± 0.62) × 10^{8}	(7.50 ± 2.57) × 10^{7}

Each value is the mean ± SD (n=3). Community nomenclature is indicated as follows: A: andosol (ADS) or G: gray lowland soil (GLS), and N: non-flooded or F: flooded conditions, and W: microcosm with the USDA110^{T} wild–type or D: microcosm with the USDA110Δ*nosZ* variant. Additionally, High: high incubation temperature (H: 30˚C), Medium: medium incubation temperature (M: 25˚C), Low: low incubation temperature (L: 20˚C). Although these incubations were conducted using sterile soil, and sterile water was supplied where necessary, the water supply was conducted under semi-sterile conditions. These cfu values are indicated as references.

a,b,c: different characters indicate significant differences among incubation periods (0, 30, 60 d) in the treatment (Bonferroni, p<0.05).

Table S2 Abundance (%) of bradyrhizobia in soil microcosms under different temperature and flooding conditions

		0day	IP[1]	30day	IP[1]	T[2]	F[3]	60day	IP[1]	T[2]	F[3]
ANW_H	U6	44.76 ± 9.84		32.07 ± 1.49		x		31.85 ± 3.61			
	U76	27.11 ± 12.02		42.28 ± 4.70			*	48.02 ± 6.72			
	U110	12.71 ± 7.34		23.13 ± 2.79			*	16.97 ± 7.31			*
	U123	15.42 ± 9.35		2.52 ± 0.94		x		3.16 ± 1.08			
ANW_M	U6	44.76 ± 9.84		48.37 ± 2.52		y	*	41.09 ± 7.33			
	U76	27.11 ± 12.02		30.29 ± 1.18			*	37.24 ± 1.75			
	U110	12.71 ± 7.34		15.08 ± 1.21			*	17.58 ± 6.38			*
	U123	15.42 ± 9.35		6.25 ± 0.47		y		4.08 ± 0.74			
ANW_L	U6	44.76 ± 9.84		37.21 ± 2.73		xy		35.41 ± 2.44			
	U76	27.11 ± 12.02		37.64 ± 2.37			*	43.11 ± 3.08			*
	U110	12.71 ± 7.34		16.44 ± 3.27			*	16.49 ± 5.91			*
	U123	15.42 ± 9.35		8.72 ± 1.71		y	*	4.98 ± 0.64			*
AFW_H	U6	44.76 ± 9.84		16.93 ± 7.00				14.07 ± 12.21			
	U76	27.11 ± 12.02		24.30 ± 3.13			*	47.61 ± 17.66			
	U110	12.71 ± 7.34	a	54.44 ± 6.14	b		*	36.25 ± 6.22	b		*
	U123	15.42 ± 9.35		4.33 ± 1.54				2.06 ± 0.24			
AFW_M	U6	44.76 ± 9.84		35.35 ± 3.05			*	31.04 ± 0.59			
	U76	27.11 ± 12.02		14.03 ± 1.32			*	28.62 ± 7.59			
	U110	12.71 ± 7.34	a	45.87 ± 3.58	b		*	36.97 ± 8.87	ab		*
	U123	15.42 ± 9.35		4.75 ± 1.61				3.37 ± 1.17			
AFW_L	U6	44.76 ± 9.84		40.54 ± 2.71				40.42 ± 5.00			
	U76	27.11 ± 12.02		16.88 ± 2.28			*	20.34 ± 3.80			*
	U110	12.71 ± 7.34	a	37.70 ± 3.57	b		*	36.52 ± 6.16	b		*
	U123	15.42 ± 9.35		4.88 ± 0.79			*	2.72 ± 0.96			*
GNW_H	U6	7.89 ± 5.49		19.82 ± 13.55				25.18 ± 7.02			*
	U76	7.57 ± 0.27	a	37.69 ± 7.17	ab			39.08 ± 4.63	b		
	U110	57.84 ± 5.70	a	31.57 ± 9.19	ab		*	31.27 ± 5.81	b		*
	U123	26.70 ± 0.39	a	10.92 ± 3.32	b	x	*	4.47 ± 2.67	b	x	
GNW_M	U6	7.89 ± 5.49	a	38.17 ± 3.75	b		*	35.41 ± 1.13	b		*
	U76	7.57 ± 0.27	a	16.15 ± 2.10	a			23.49 ± 1.66	b		
	U110	57.84 ± 5.70	a	18.44 ± 1.98	b		*	17.89 ± 3.13	b		*
	U123	26.70 ± 0.39		27.24 ± 0.37		y	*	23.22 ± 1.85		y	*
GNW_L	U6	7.89 ± 5.49	a	29.82 ± 2.66	b			23.37 ± 6.25	ab		
	U76	7.57 ± 0.27	a	16.49 ± 5.64	ab			30.89 ± 2.64	b		
	U110	57.84 ± 5.70	a	17.85 ± 3.66	b		*	19.09 ± 3.31	b		*
	U123	26.70 ± 0.39		35.84 ± 9.44		xy	*	26.64 ± 0.66		y	*
GFW_H	U6	7.89 ± 5.49		10.02 ± 1.29		x		4.70 ± 4.00		x	*
	U76	7.57 ± 0.27		28.64 ± 9.68				24.78 ± 11.35			
	U110	57.84 ± 5.70		58.63 ± 9.68			*	69.00 ± 15.60			*
	U123	26.70 ± 0.39	a	2.71 ± 0.19	b	x	*	1.52 ± 0.27	b	x	
GFW_M	U6	7.89 ± 5.49		15.63 ± 8.23		xy	*	14.65 ± 2.37		xy	*
	U76	7.57 ± 0.27	a	12.18 ± 0.96	b			23.53 ± 8.79	ab		
	U110	57.84 ± 5.70		64.28 ± 8.36			*	57.40 ± 10.10			*
	U123	26.70 ± 0.39	a	7.92 ± 3.22	b	xy	*	4.42 ± 0.53	b	y	*
GFW_L	U6	7.89 ± 5.49		26.45 ± 2.12		y		21.81 ± 5.21		y	
	U76	7.57 ± 0.27		12.47 ± 2.95				26.32 ± 4.97			
	U110	57.84 ± 5.70		53.57 ± 2.44			*	45.74 ± 6.80			*
	U123	26.70 ± 0.39	a	7.51 ± 0.67	b	y	*	6.13 ± 2.44	b	y	*

		0day	IP[1]	30day	IP[1]	T[2]	F[3]	60day	IP[1]	T[2]	F[3]
AND_H	U6	51.81 ± 15.99		59.06 ± 3.81			*	53.61 ± 3.51			*
	U76	16.32 ± 7.74		32.14 ± 0.52		x	*	34.08 ± 5.08			*
	U110	7.86 ± 6.05		1.02 ± 0.50				1.43 ± 0.97			
	U123	24.01 ± 3.60	a	7.78 ± 4.39	b			10.87 ± 3.38	b		
AND_M	U6	51.81 ± 15.99		58.95 ± 3.35				56.54 ± 2.04			*
	U76	16.32 ± 7.74		23.15 ± 1.73		y		29.97 ± 0.52			*
	U110	7.86 ± 6.05		0.54 ± 0.23			*	1.07 ± 0.75			
	U123	24.01 ± 3.60		17.37 ± 2.13				12.42 ± 1.60			
AND_L	U6	51.81 ± 15.99		53.74 ± 4.11			*	53.34 ± 3.92			
	U76	16.32 ± 7.74		26.04 ± 1.01		y	*	29.95 ± 3.99			
	U110	7.86 ± 6.05		0.46 ± 0.19				0.96 ± 0.34			
	U123	24.01 ± 3.60		19.76 ± 2.99				15.76 ± 2.28			*
AFD_H	U6	51.81 ± 15.99	a	38.18 ± 5.58	ab	x	*	18.60 ± 10.70	b		*
	U76	16.32 ± 7.74	a	44.98 ± 4.17	b	x	*	63.72 ± 9.27	b	x	*
	U110	7.86 ± 6.05		11.71 ± 4.98				12.14 ± 6.19			
	U123	24.01 ± 3.60	a	5.12 ± 0.94	b			5.54 ± 2.97	b		
AFD_M	U6	51.81 ± 15.99		48.80 ± 5.50		x		29.22 ± 8.37			*
	U76	16.32 ± 7.74	a	26.59 ± 8.72	ab	x		48.56 ± 1.37	b	x	*
	U110	7.86 ± 6.05		7.64 ± 2.64			*	13.02 ± 7.81			
	U123	24.01 ± 3.60	a	16.98 ± 3.64	ab			9.20 ± 0.79	b		
AFD_L	U6	51.81 ± 15.99		70.55 ± 1.46		y	*	49.97 ± 7.13		ns	
	U76	16.32 ± 7.74		15.34 ± 3.01		y	*	33.20 ± 9.56		y	
	U110	7.86 ± 6.05		1.17 ± 0.55				7.66 ± 3.16			
	U123	24.01 ± 3.60	a	12.95 ± 4.16	ab			9.17 ± 1.08	b		*
GND_H	U6	8.77 ± 5.49	a	36.39 ± 11.75	ab	xy		36.78 ± 4.65	b		
	U76	16.09 ± 0.27		32.09 ± 7.77				40.46 ± 6.89			
	U110	49.39 ± 5.70	a	20.82 ± 9.29	b			16.73 ± 2.99	b	x	
	U123	25.74 ± 0.39	a	10.70 ± 4.05	ab	x		6.02 ± 3.35	b	x	
GND_M	U6	8.77 ± 5.49	a	51.08 ± 4.10	b	x		29.90 ± 14.82	ab		
	U76	16.09 ± 0.27		15.42 ± 0.67				34.60 ± 7.73			
	U110	49.39 ± 5.70	a	1.53 ± 0.42	b			6.65 ± 6.92	b	xy	
	U123	25.74 ± 0.39		31.97 ± 3.60		y		28.85 ± 1.24		y	
GND_L	U6	8.77 ± 5.49	a	37.74 ± 2.48	b	y		32.67 ± 4.25	b		
	U76	16.09 ± 0.27		19.19 ± 7.02				28.60 ± 4.28			
	U110	49.39 ± 5.70	a	1.30 ± 0.37	b		*	1.43 ± 0.39	b	y	*
	U123	25.74 ± 0.39		41.77 ± 6.27		y		37.29 ± 4.59		y	
GFD_H	U6	8.77 ± 5.49		25.25 ± 3.21				16.49 ± 12.12			
	U76	16.09 ± 0.27	a	40.74 ± 5.59	b	x		43.34 ± 8.63	ab		
	U110	49.39 ± 5.70		31.39 ± 3.48		x		33.40 ± 12.81			
	U123	25.74 ± 0.39	a	2.62 ± 1.00	b			6.77 ± 5.51	ab		
GFD_M	U6	8.77 ± 5.49		50.98 ± 29.27				20.48 ± 11.54			
	U76	16.09 ± 0.27		12.89 ± 7.57		y		38.36 ± 16.56			
	U110	49.39 ± 5.70	a	13.43 ± 4.94	b	y		22.66 ± 14.89	ab		
	U123	25.74 ± 0.39		22.69 ± 17.52				18.50 ± 14.14			
GFD_L	U6	8.77 ± 5.49		52.98 ± 18.61				29.25 ± 18.14			
	U76	16.09 ± 0.27	ab	10.78 ± 3.72	a	y		24.79 ± 3.49	b		
	U110	49.39 ± 5.70	a	7.09 ± 1.91	b	y	*	7.55 ± 2.01	b		*
	U123	25.74 ± 0.39		29.15 ± 15.30				38.42 ± 20.81			

Each value is the mean ± SD (n=3). Community nomenclature is indicated as follows: A: andosol (ADS) or G: gray lowland soil (GLS), and N: non-flooded or F: flooded conditions, and W: microcosm with the USDA110^T wild–type or D: microcosm with the USDA110Δ*nosZ* variant. Additionally, High: high incubation temperature (H: 30˚C), Medium: medium incubation temperature (M: 25˚C), Low: low incubation temperature (L: 20˚C).

[1]IP(a,b): different characters indicate significant differences among incubation periods (0, 30, 60 d) in the treatment (Bonferroni, $p<0.05$).

[2]T(x,y): different characters indicate significant differences among incubation temperatures (H, M, L) in the treatment (Bonferroni, $p<0.05$).

[3]F(*): asterisk indicates a significant difference between flooded and non-flooded conditions in the treatment (Welch's t-test, $p<0.05$).
